# Acute consequences of a unilateral VIIIth nerve transection on vestibulo-ocular and optokinetic reflexes in *Xenopus laevis* tadpoles

**DOI:** 10.1007/s00415-020-10205-x

**Published:** 2020-09-11

**Authors:** Parthena Soupiadou, Clayton Gordy, Michael Forsthofer, Rosario Sanchez-Gonzalez, Hans Straka

**Affiliations:** 1 Department Biology II, Ludwig-Maximilians-University Munich, Großhaderner Str. 2, 82152 Planegg, Germany; 2 Graduate School of Systemic Neurosciences, Ludwig-Maximilians-University Munich, Großhaderner Str. 2, 82152 Planegg, Germany

**Keywords:** Vestibulo-ocular reflex, Semicircular canal, Extraocular motoneurons, Eye movements, Unilateral labyrinthectomy, Homeostatic plasticity, Optokinetic reflex

## Abstract

Loss of peripheral vestibular function provokes severe impairments of gaze and posture stabilization in humans and animals. However, relatively little is known about the extent of the instantaneous deficits. This is mostly due to the fact that in humans a spontaneous loss often goes unnoticed initially and targeted lesions in animals are performed under deep anesthesia, which prevents immediate evaluation of behavioral deficits. Here, we use isolated preparations of *Xenopus laevis* tadpoles with functionally intact vestibulo-ocular (VOR) and optokinetic reflexes (OKR) to evaluate the acute consequences of unilateral VIIIth nerve sections. Such in vitro preparations allow lesions to be performed in the absence of anesthetics with the advantage to instantly evaluate behavioral deficits. Eye movements, evoked by horizontal sinusoidal head/table rotation in darkness and in light, became reduced by 30% immediately after the lesion and were diminished by 50% at 1.5 h postlesion. In contrast, the sinusoidal horizontal OKR, evoked by large-field visual scene motion, remained unaltered instantaneously but was reduced by more than 50% from 1.5 h postlesion onwards. The further impairment of the VOR beyond the instantaneous effect, along with the delayed decrease of OKR performance, suggests that the immediate impact of the sensory loss is superseded by secondary consequences. These potentially involve homeostatic neuronal plasticity among shared VOR-OKR neuronal elements that are triggered by the ongoing asymmetric activity. Provided that this assumption is correct, a rehabilitative reduction of the vestibular asymmetry might restrict the extent of the secondary detrimental effect evoked by the principal peripheral impairment.

**Electronic supplementary material:**

The online version of this article (10.1007/s00415-020-10205-x) contains supplementary material, which is available to authorized users.

## Introduction

Unilateral loss of peripheral vestibular function causes severe and incapacitating symptoms [1]. These emerging deficits derive from either an impairment of inner ear structures or are commonly observed following damage to the statoacoustic (VIIIth) nerve. Well characterized impairments of this nerve, such as neuritis, schwannoma, or surgical transection each provide some degree of peripheral vestibular loss that is usually accompanied by vertigo, dizziness, oscillopsia, and various cognitive deficits in orientation and navigation [2]. Furthermore, pathological motor reactions such as a nystagmus or postural asymmetries also occur following VIIIth nerve disfunction [2–7]. Human patients with impaired vestibular function presenting to a clinician, however, have usually suffered from such symptoms for many days prior to clinical assessment, with the more immediate and most severe impairments having often vanished. In addition, the presented symptoms are often superimposed with and influenced by, alterations that derive from vestibular compensation, a plasticity process thought to ameliorate lesion-induced deficits [1], which often encumbers diagnostic effectiveness.

Experimental reproduction of vestibular deficits in animal models also suffer from a general lack of detailed knowledge on the magnitude, variety, and progression of acute symptoms that appear instantaneously after an induced loss of peripheral vestibular function. This is mostly due to the fact that any peripheral vestibular lesion has to be performed in deeply anesthetized and analgesically treated animals that require a post-surgical recovery period until the behavioral impairments can be faithfully evaluated. During this period, the activity of the nervous system is considerably attenuated and thus unable to appropriately express immediate functional deficits [8]. Compared to typical patients with a vestibular syndrome that are seen by a clinician days and weeks after the incident, a planned tumor surgery of the VIIIth nerve [9] or a comparable experimental manipulation, e.g. in rabbits [10] or mice [11] are currently the closest conditions that allow an evaluation of the acute stage after a vestibular lesion. Nonetheless, all these studies suffer from the unavoidable temporal lag between the surgical lesion and the fully awake state of an animal, which is required to estimate the full spectrum of immediate behavioral consequences.

The difficulties of evaluating acute motor impairments after a unilateral peripheral vestibular nerve lesion can be circumvented, however, by employing the amphibian *Xenopus laevis* as a model system. In particular, an isolated in vitro whole-head preparation of *Xenopus laevis* tadpoles with intact sensory organs (eyes, inner ears) and motor effector organs (eye muscles) to execute visuo-vestibular motion-evoked eye movements allow an in vivo-like approach and manipulations under in vitro conditions [12]. Specifically, the isolated nature of the in vitro preparation allows a rapid surgical transection of the VIIIth nerve under visual guidance in the absence of anesthesia, providing the necessary condition to characterize and quantify the instantaneous behavioral consequences of a unilateral vestibular loss. To evaluate the impact of a VIIIth nerve transection, the current study directly assessed the behavioral output from ocular motor centers, where both vestibular and visual information converges. Accordingly, spontaneous eye position changes as well as vestibulo-ocular reflex (VOR) and visual image motion-evoked optokinetic reflex (OKR) performance during separate or combined horizontal visuo-vestibular motion stimulation [13, 14] were assessed.

In this study, static and motion-evoked eye movements were recorded with an infrared video camera prior to and immediately after a unilateral transection of the VIIIth nerve. The gain and phase magnitudes of evoked eye movements at four time points, over a period of up to 5 h postlesion, were analyzed to estimate the acute consequences of the lesion and to evaluate the plasticity of the ocular motor behavior.

## Material and methods

### Animals and experimental preparation

*Xenopus laevis* tadpoles of either sex (*n* = 7) at developmental stages 53–55 [15] were obtained from the in-house animal breeding facility at the Biocenter-Martinsried of the Ludwig-Maximilians-University Munich. Tadpoles were maintained in tanks with non-chlorinated water (17–18 °C) at a 12/12 light/dark cycle. Experiments were performed in vitro on semi-intact preparations and comply with the "Principles of animal care", publication No. 86-23, revised 1985 of the National Institute of Health. Permission for these experiments was granted by the Regierung von Oberbayern (ROB-55.2–2532.Vet_03-17-24).

**Fig. 1:**
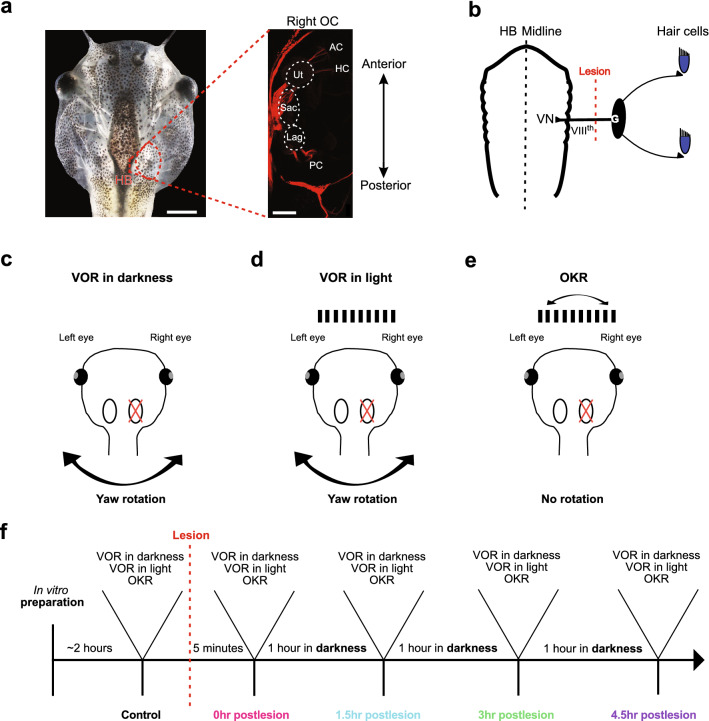
Experimental paradigm for evaluating acute consequences of a unilateral VIIIth nerve section on eye movements. **a** Isolated head preparation of a stage 55 *Xenopus laevis* tadpole with functional eyes, eye muscles, inner ears and neuronal circuits for ocular motor behavior; the inset on the right illustrates afferent innervation patterns of vestibular endorgans after tracer placement (Tetramethylrhodamine) into the vestibular nuclei. **b** Schematic of the vestibular hair cell—hindbrain vestibular nucleus (VN) connection depicting the VIIIth nerve, ganglion of *Scarpa* (G) and site of the postganglionic nerve section (lesion). **c**–**e** Schematics illustrating the three experimental paradigms used to evaluate the impact of the unilateral lesion: vestibulo-ocular reflex in darkness (VOR in darkness; **c**), in light (VOR in light; **d**) and optokinetic reflex (OKR; **e**). **f** Flow chart illustrating the temporal sequence of prelesional control recordings, nerve transection, and postlesional recordings of visuo-vestibular motion-evoked eye movements. *AC, PC, HC* anterior, posterior vertical, horizontal semicircular canal, *HB* hindbrain, *Lag* lagena, *OC* otic capsule, *Sac* saccule, *Ut* utricle. Scale bars in a are 2 mm and 50 µm, respectively

Tadpoles were anesthetized in 0.05% 3-aminobenzoic acid ethyl ester methanesulfonate at room temperature (MS-222; Pharmaq Ltd. UK) for 3 min, transferred to ice-cold frog Ringer solution (75 mM NaCl, 25 mM NaHCO_3_, 2 mM CaCl_2_, 2 mM KCl, 0.1 mM MgCl_2_, and 11 mM glucose, pH 7.4), decapitated at the level of the upper spinal cord (Fig. 1a) and pinned to a Sylgard base with the ventral side up to remove the lower jaw and viscera under visual control. The skin was removed from the remaining tail and all spinal nerves were severed to prevent swim-related contractions of the most anterior axial muscles. The cartilaginous skull was opened from dorsal and the forebrain was removed. The hindbrain entrances of both VIIIth nerves were exposed by removal of connective tissue above and around the brain, as well as by removal of the choroid plexus overlaying the fourth ventricle. However, the remaining central nervous system, visual, and vestibular sensory periphery with afferent connections, and extraocular motor nerves were functionally preserved. This allowed the recording of eye movements during application of visual and vestibular motion stimuli. Following, the preparations were allowed to recover for ~ 2 h at 17 °C before commencing with the recording session [16]. During a recording session, preparations were mechanically secured in the center of a Sylgard-lined chamber and continuously superfused with oxygenated (Carbogen: 95% O_2_, 5% CO_2_) Ringer solution at a constant temperature of 17.5 ± 0.5 °C.

Because of the maintained neuronal innervation of the extraocular muscles, the isolated preparation allowed the activation of eye movements by vestibular and visual motion stimulation. Natural activation of the vestibular endorgans was performed with a six degrees of freedom motion stimulator (PI H-840, Physik Instrumente, Karlsruhe, Germany). Vestibular motion stimuli consisted of sinusoidal horizontal rotations at 0.5 Hz and positional excursion of ± 10° that generated a peak velocity of ± 31.4°/s. Visual pattern motion was provided in an open-loop virtual reality setting formed by an open cylindrical screen, encompassing 275° with a diameter of 8 cm and a height of 5 cm. Three digital light processing (DLP) video projectors (Aiptek V60), installed in 90° angles to each other were affixed to the table surrounding the screen and projected a visual pattern at a refresh rate of 60 Hz onto the screen [14]. The pattern consisted of equally spaced, vertical, black and white stripes with a spatial size of 16°/16°. The pattern motion consisted of horizontal sinusoidal oscillations at 0.2 Hz and positional excursions of ± 10° (± 12.6°/s peak velocity). For all experiments, the Sylgard-lined recording chamber with the affixed preparation was placed in the center of the cylindrical screen that co-aligned with the vertical rotation axis of the motion stimulator. Visual and vestibular motion stimuli were applied either separately or in combination to evoke a VOR in darkness, VOR in light (in the presence of world-stationary vertical black and white stripes) or an OKR (Fig. 1c–e). The temporal sequence of prelesional control recordings, nerve transection, and postlesional recordings of visuo-vestibular motion-evoked eye movements is illustrated in the flow chart of Fig. 1f.

Eye movements were recorded non-invasively with an infrared video camera (Grasshopper mono, Point Grey Research Inc., Canada) and a zoom objective (Optem Zoom 70XL, Qioptiq Photonics GmbH & Co. KG, Germany) with an adequate lens (M25 × 0.75 + 0.25) as previously described [16]. This system was mounted on top of the experimental setup to visualize the motion of both eyes from above during visual and vestibular motion stimulation at a video capture frame rate of 30 Hz with FlyCap2 software (v2.3.2.14.). Eye motion profiles and parameters were extracted from the captured video sequences using a custom video-processing algorithm written in C++ (for details see [17]). To calculate the motion of the eyes, an ellipse was drawn around each eyeball and the angle between the major axis of the ellipse and the longitudinal axis of the head was calculated in each frame of a given video sequence. Based on the frame rate (30 Hz), the change in eye position over time was then computed.

### Unilateral surgical transection of the VIIIth nerve

The plain visibility of the central nervous system (CNS) and cranial nerve roots facilitated a targeted transection of the right VIIIth nerve by cutting the nerve between its entrance into the hindbrain and the inner wall of the otic capsule with a microscissor under direct visual control. All procedures were completed under a binocular microscope, which allowed precise and complete transection of the entire nerve bundle with a single cut. Great care was taken to not damage the hindbrain, the otic capsule or other cranial nerves traversing ventrally in proximity to the VIIIth nerve. Based on the site of the transection, between the medial wall of the otic capsule (Fig. 1b) and the entry into the hindbrain, the lesion was postganglionic and accordingly disconnected the ganglion of *Scarpa* from the brain [18].

### Data analysis

Eye and stimulus positions were recorded in Spike2 (Cambridge Electronic Design, UK) for off-line analysis. For all subsequent analysis, eye and stimulus position data was resampled to 100 Hz using linear interpolation, and eye position traces were smoothed with a 0.05 s time constant with built-in Spike2 functions. Eye positions over a single head/table or visual image motion cycle were obtained from the recorded data using a custom Spike2 script for single-cycle extraction and consolidated with a custom script written in Python 3. Average responses were calculated from 20–30 cycles. Respective magnitudes were computed from peak-to-peak amplitudes of “successful” VOR cycles (see “Results”). The phase relation of motion-induced eye movements with respect to the table position was obtained by comparing the timing of the average response peak with the timing of the maximal stimulus position or visual motion pattern deflection. To assess if the motion of both eyes was conjugated, bilateral eye positions were exported from Spike2 and plotted against each other. While exporting, a lower sampling rate of 10 Hz was used to avoid oversaturation of the plots. If data points did not have a corresponding value at each sampling interval, the nearest temporal point was used instead. To assess static eye position, average resting eye positions over 10 s were extracted from Spike2 prior to starting the first recording session in darkness. The data were further processed and analyzed statistically using Prism (GraphPad Software, Inc, USA). Responses were normalized and averaged (± SD; standard deviation) for comparison. Statistical differences were calculated with the Friedman test and Dunn’s multiple comparisons test (non-parametric, paired data; Prism, GraphPad Software, Inc, USA). Statistical analysis with the Friedman test will be reported only in the text while Dunn’s test will be reported also in the figures and/or legends.

### Dextran amine dye tracings

Fluorescent visualization of the VIIIth nerve was used to provide a pre-experimental visual reference of the distal entrance site of the nerve, ganglion cell bodies, and peripheral neurites within the otic capsule for subsequent experimental procedures requiring transection of unlabeled nerves. Tetramethylrhodamine (543 nm; 3000 MW; Invitrogen, D3308) crystals were dissolved until a viscous solution was produced. Pins affixed to glass micropipettes were placed in this viscous tracer solution, coated with a high concentration of tracer and inserted unilaterally into the vestibular nuclei of the hindbrain in an in vitro preparation. Tracer spread to neighboring structures was carefully avoided. The preparation was then transferred into 200 ml freshly-oxygenated Ringer solution and incubated at 17 °C for 24 h (*n* = 1). Thereafter, the preparation was fixed in 4% paraformaldehyde (PFA) for 24 h and cleared using the uDISCO method [19]. In brief, the preparation was serially incubated in 30, 50, 70, 80, 90 and 96 vol% tert-butanol (2 h each; Sigma, 360538), and then cleared in a mixture of benzyl alcohol (Sigma, 24122-M), benzyl benzoate (Sigma, W213802) and diphenyl ether (Alpha Aesar, A15791) corresponding to BABB-D15 according to Pan et al. [19]. Subsequently, the tissue was mounted and coverslipped in BABB-D15 using a custom metal spacer before imaging on an Olympus Fluoview confocal microscope (FV 10-ASW 2.1 software).

## Results

The acute effects of unilateral vestibular nerve sections on static eye position and visuo-vestibular motion-induced eye movements were evaluated immediately and up to 5 h postlesion. The analysis of ocular motor performance in prelesional conditions for each experimental animal allowed reliable quantification of the behavioral impairment prior to and over the first few hours after the unilateral loss of peripheral vestibular function. Prelesional performance was first assessed to determine the range of unmanipulated responses and was subsequently followed by identical assessment after the VIIIth nerve lesion.

### Prelesional eye position and motion dynamics during visuo-vestibular stimulation

#### Resting eye position and stimulus-evoked eye movements

**Fig. 2:**
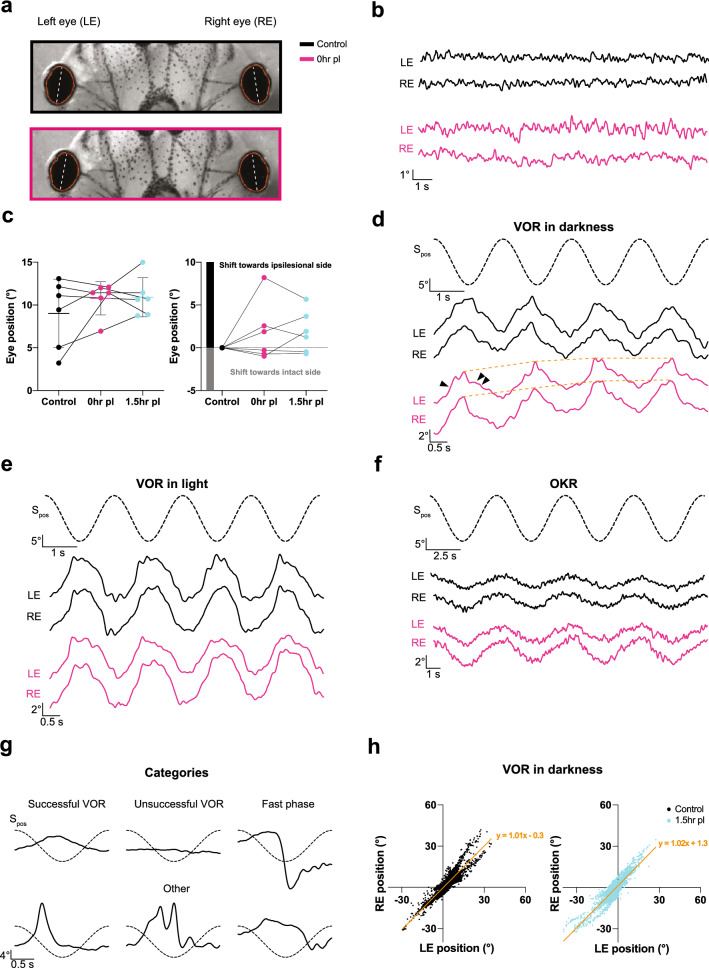
Spontaneous and visuo-vestibular motion-evoked eye movements. **a, b** Infrared images (**a**) and static eye position of the left (LE) and right (RE) eye (**b**) before (upper image in **a**, black trace in **b**) and immediately after (0 h) transection of the right VIIIth nerve (lower image in **a**; magenta trace in **b**); dashed white lines in **a** indicate the major axis of the oval-shaped eyes, used to measure eye position and evoked motion. **c** Dot and whisker plot of the absolute eye position relative to the longitudinal body axis (left) and following subtraction of the prelesional eye position (right) in controls, immediately (0 h, magenta) and 1.5 h after transection of the right VIIIth nerve (cyan) in darkness. **d-f** Examples of movements of the left and right eye during four consecutive cycles of horizontal sinusoidal rotation of the head/table (0.5 Hz; ± 31.4°/s) in darkness (VOR in darkness; **d**), in light (VOR in light; **e**), and of horizontal sinusoidal motion (0.2 Hz; ± 12.6°/s) of a vertical black and white striped pattern (OKR; **f**) before (black traces) and immediately after (0 h) transection of the right VIIIth nerve (magenta traces); dashed sinusoids represent stimulus position profiles (S_pos_); arrowheads indicate eye movements evoked by head/table motion towards the intact (single arrowhead) and ipsilesional (two arrowheads) side; dotted orange line in **d** indicates the gradual shift in eye position towards the ipsilesional side with each rotation cycle. **g** Qualitative categorization of eye movements evoked by vestibular motion stimulation labeled as “successful”, “unsuccessful”, “fast phases” and “other”; dashed sinusoids represent the stimulus position (± 10°) profiles of the head/table motion cycle at 0.5 Hz. **h** Conjugation correlation plots of the position of the left and right eye during horizontal sinusoidal head/table rotation (VOR in darkness) before (black) and 1.5 h (cyan) after transection of the right VIIIth nerve; note that the position of both eyes is closely correlated indicating strict conjugation during the horizontal angular VOR before and after the lesion

The eyes of *Xenopus laevis* tadpoles at mid-larval stages have a lateral position with a slightly nasal orientation of 5–10° relative to the longitudinal head/body axis (Figs. 1a, 2a) with an ocular motor range of ~20°, estimated by systematic analysis of the OKR performance [14]. In darkness, in the absence of visuo-vestibular motion stimulation, the position of both eyes remained relatively stable except for very small (~1°), horizontal oscillations (Fig. 2b) despite variability in absolute resting position between animals (Fig. 2c; black). The variability of the resting eye position between preparations likely derives from the combinatorial influence of potentially inconsistent horizontal placements of the latter within the recording chamber, variations in the electronic detection of the oval-shaped eyes by the tracking software and development-related differences of the eye position between animals at stage 53–55. Despite this variability, a consistent and most notable aspect was the absence of scanning saccadic eye movements, with the exception of infrequent locomotion-related fast horizontal eye deflections [20]. During prelesional control conditions, horizontal sinusoidal rotation in darkness (0.5 Hz; ± 31.4°/s peak velocity; *n* = 6) evoked movements of both eyes that were directed in phase-opposition to the stimulus, indicative of a functional angular VOR (black traces in Fig. 2d). Eye movements during most cycles of the motion stimulus had waveforms and dynamics that matched well with the expectations of a “successful” VOR in *Xenopus* tadpoles (Fig. 2d,g) [13]. Other cyclic response types, although fewer, appeared to be “unsuccessful” attempts, as characterized mostly by negligible responses, “fast phases” of variable kinetics and magnitudes [14] or were designated as “other” because of uncertain classification that did not allow reliable assignment to a particular category (Fig. 2g; Supplementary Fig. 2a). The common denominator of the latter class was an eye motion peak velocity that exceeded, in part considerably, stimulus peak velocity and thus did not meet the criteria of a VOR slow phase, which by definition can maximally adopt stimulus motion magnitudes [21].

**Fig. 3:**
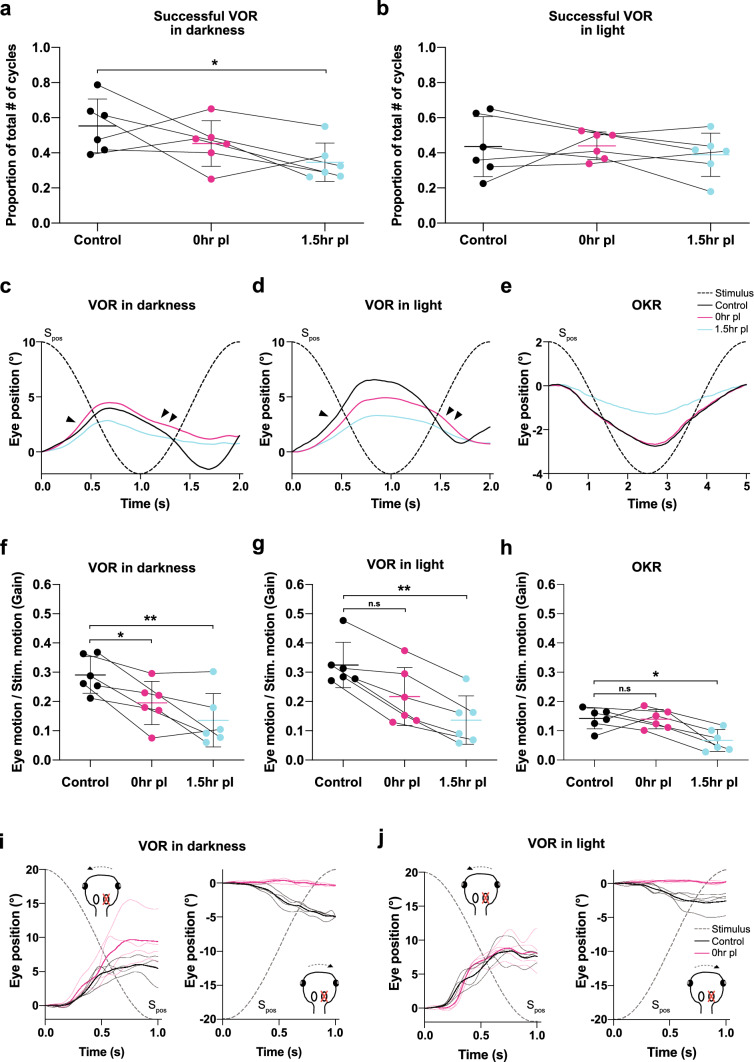
Immediate effects of a unilateral VIIIth nerve section on eye movement dynamics. **a**, **b** Relative proportion of “successful” VOR responses before (black), immediately (0 h, magenta) and 1.5 h postlesion (cyan) during sinusoidal rotation in darkness (**a**) and in light (**b**). **c**–**e** Averages of “successful” VOR responses in darkness (**c**), in light (**d**) and OKR responses (**e**) over a single sinusoidal stimulus motion cycle; averages are the mean of the responses from all preparations (*n* = 6), respectively; dashed sinusoids represent the stimulus position profile (*S*_pos_); arrowheads indicate eye movements evoked by head/table motion towards the intact (single arrowhead) and ipsilesional (two arrowheads) side, respectively. **f**–**h** Response gains (eye motion / stimulus motion) before, at 0 h and 1.5 h after the lesion for the VOR in darkness (**f**), in light (**g**) and the OKR (**h**). **i**–**j** Representative example traces of sinusoidal VOR eye movements over the first half cycle during head/table motion in darkness (**i**) and in light (**j**) directed towards the intact (left) and ipsilesional side (right) before (black traces) and immediately after the VIIIth nerve section (0 h, magenta traces); thin and thick lines represent individual and average responses, respectively. *p < 0.05; **p < 0.01; Dunn’s multiple comparisons test with respect to control values

A principally analogous pattern with similar dynamics of eye movements was evoked during horizontal sinusoidal motion stimulation in the presence of a black and white vertically striped, world-stationary visual pattern (VOR in light; black traces in Fig. 2e). The majority of eye movements were again designated as “successful” VOR, with responses that were generally more robust than the VOR in darkness (Figs. 2g, 3d). Likely due to the concurrent effect of the world-stationary visual pattern, “unsuccessful” attempts to stabilize gaze were almost completely absent (Supplementary Fig. 2b). In contrast, eye movements classified as “fast-phases” or “other” were found in similar proportions as during application of a sinusoidal motion stimulus in darkness. This indicates that vestibular motion stimulation evokes a qualitatively similar VOR in larval *Xenopus* under both illumination conditions, although, eye movements produced in the presence of a world-stationary black and white striped pattern were more robust. This enhanced robustness most likely derived from the synergistic performance of vestibular and optokinetic reflexes during turntable motion and concurrent relative motion of the visual pattern. To isolate the contribution of visual motion-induced eye movements during activation of the VOR in light, the OKR was separately elicited by visual pattern motion in the absence of turntable rotation. Horizontal sinusoidal motion of black and white vertical stripes (0.2 Hz; ± 12.6°/s peak velocity; *n* = 6) evoked oscillatory movements of both eyes that aimed at following the stimulus (Fig. 2f), in correspondence with the spatio-temporal dynamics of a functional OKR in *Xenopus* tadpoles (see [13]). The responses were robust with no resetting “fast phases”. As described for the resting eye position in darkness (Fig. 2b), the eyes also remained relatively stationary in light, except for small irregular horizontal oscillations with magnitudes of ~1° (not shown).

#### Eye motion performance

All three different visuo-vestibular motion stimulus paradigms evoked horizontal movements of both eyes that were directionally coordinated (Fig. 2d–f). The extent of coordinated conjugation for the left and right eye was quantitatively evaluated by calculating the bilateral response coordination from 27–30 cycles of the VOR (in darkness), omitting twitch-like eye movements. Plotting eye position magnitudes of the left eye (*x*-axis) versus the right eye (*y*-axis) confirmed that the positions of the two eyes during motion stimulation were strongly correlated with each other (VOR in darkness: *λ* = 1.015; *r*^*2*^ = 0.8567; *n* = 6), yielding a slope close to 1 (Fig. 2h, left panel). This close correspondence indicated a strict conjugation of both eyes during stimulus-triggered motion, despite the lack of a fovea in these animals and the rather lateral position of the eyes. After averaging across multiple cycles (Fig. 3c–e), gain values *re* stimulus were calculated for “successful” VOR and OKR responses, respectively (Fig. 3f–h). Under control conditions, i.e., prior to the unilateral section of the VIIIth nerve, this analysis yielded a gain value (eye motion/stimulus motion) for the VOR in darkness of 0.29 ± 0.1, for the VOR in light of 0.32 ± 0.08 and for the OKR during sinusoidal motion stimulation of 0.14 ± 0.04 (all values are mean ± SD; *n* = 6, respectively). Peak responses *re* stimulus position had phase leads of − 54° (− 54.3° ± 12.34°) and − 29° (− 29.4° ± 18.2°) for the VOR in darkness and in light, respectively. For the OKR, the peak response was nearly phase-aligned with stimulus position (13.4° ± 10.1°; values are mean ± SD; *n* = 6, respectively). These values complied with those reported earlier for *Xenopus* tadpoles at this developmental stage [13, 14].

### Postlesional effects following unilateral transection of the VIIIth nerve

#### Transection of the VIIIth nerve

The plain visibility of the hindbrain and cranial nerve roots allowed a rapid, targeted transection of the right VIIIth nerve by a single cut with a microscissor between the nerve entrance and *Scarpa´s* ganglion (Fig. 1b). This controlled surgical intervention ensured that no other cranial nerve traversing in close vicinity such as the abducens nerve was unintentionally harmed. Since the transections were made in vitro in the absence of neuronal activity-suppressing anesthetic agents, the consequences on eye position and evoked eye motion could be evaluated immediately after the lesion.

#### Impact on resting eye position

The impact of the unilateral peripheral vestibular impairment on resting eye position in darkness was evaluated immediately (0 h) and 1.5 h after the nerve section. Most notably, a spontaneous nystagmus, as present for instance in mammalian species [11, 22] including human patients [1], was not observed in any preparation. Instead, both eyes continued to remain relatively stable except for horizontal oscillations with similarly small magnitudes as those observed in controls (compare magenta and black traces in Fig. 2b). Even though the prelesional resting position was variable across animals (black in Fig. 2c), the average resting position of both eyes tended to shift towards the ipsilesional side immediately after the lesion (lower image in Fig. 2a at 0h; magenta in Fig. 2c). This more eccentric position was maintained at similar values at 1.5 h postlesion in 4 out of 6 animals (cyan in Fig. 2c). The postlesional alteration of individual resting eye position at the first two tested time points is more clearly illustrated in Fig. 2c, right plot, following subtraction of the individual control resting position, respectively. This average tentative shift in eye position across preparations complies with the induced asymmetry in bilateral vestibular afferent resting discharge rates following the nerve section, known to consequently cause a sustained, ipsilesionally directed excitatory drive of extraocular motoneurons [23].

#### Impact on VOR performance

VOR responses were elicited by sinusoidal head motion in darkness and in light as under control conditions. Responses were found again to be heterogenous (magenta traces in Fig. 2d,e; Supplementary Fig. 2a, b) across stimulus cycles and repetitions but in general continued to adhere to the four categories established for the control condition (see Fig. 2g). Immediately after the VIIIth nerve section, “successful” VOR responses during sinusoidal motion in darkness decreased in occurrence (magenta in Fig. 3a) but significantly more so after 1.5 h (cyan in Fig. 3a; *p* = 0.1416 Friedman test; control versus 0 h *p* = 0.5637; control versus 1.5 h *p* = 0.0433 Dunn’s multiple comparisons test). In contrast, the number of “successful” VOR responses during sinusoidal motion in light remained largely unchanged compared to control responses (magenta and cyan in Fig. 3b; *p* = 0.7402 Friedman test; control versus 0 h *p* > 0.9999; control versus 1.5 h *p* = 0.3865 Dunn’s multiple comparisons test), suggesting that the additional presence of a world-stationary black and white striped visual pattern that activates concurrent visuo-motor responses during vestibular motion stimulation in light might offset the unilateral lack of head rotational sensory signals.

In a complementary manner, the category of “unsuccessful” VOR response attempts, which was rather low in occurrence during prelesional control conditions, increased following VIIIth nerve transection (Supplementary Fig. 2a, b). While this was particularly pronounced for the VOR in darkness (*p* = 0.0009 Friedman test), the occurrence of this category was not significantly increased for VOR in light (*p* = 0.4244 Friedman test). This is likely due to the fact that this category is virtually absent in all preparations under prelesional control conditions (Supplementary Fig. 2b) and only mildly increased in reciprocal correspondence to the tendency of reduced “successful” VOR response attempts after the lesion.

At variance with the complementary postlesional alterations of the “successful” and “unsuccessful” VOR response categories, the average occurrence of “fast phases” and “other” was largely unaffected by the VIIIth nerve section during both table motion in darkness and in light (Supplementary Fig. 2a,b; Friedman test in darkness *p* = 0.6922 and in light *p* = 0.2537 for “fast phases”; *p* = 0.8781 in darkness and *p* = 0.4247 in light for “other”). The lack of an increase in the number of fast phases during motion stimulation also complies with the absence of a VIIIth nerve lesion-induced nystagmus during static head position both in light and in darkness (see above). The unchanged frequency of occurrence of eye movements during motion stimulation designated as “other” before and after the nerve section (Supplementary Fig. 2a,b) suggests that this category of jerky ocular motor behavior is independent of vestibular signals and potentially driven by spontaneous episodes of activity in brainstem or spinal locomotor circuits [20].

#### Impact on VOR response gain and phase

An VIIIth nerve section appeared to cause the evoked cyclic eye movements during sinusoidal rotation in darkness to decrease in magnitude (magenta traces in Fig. 2d) immediately after the unilateral loss of vestibular signals (0 h). The reduced responses of the VOR, although rather variable between preparations, yielded an average gain of 0.20 ± 0.10 (mean ± SD; *n* = 6) as indicated by the mean responses over a single motion cycle (Fig. 3c, f). The bidirectional eye motion components of the VOR during horizontal sinusoidal motion stimulation, however, were differentially affected. Instead of symmetric responses in both head motion directions (Fig. 2d black traces), eye movements evoked by rotation towards the side of the lesion became considerably slower and smaller in magnitude (magenta traces; two arrowheads in Fig. 2d). In contrast, eye movements evoked by rotation towards the intact side appeared to be unchanged compared to controls in terms of dynamics and amplitude (magenta traces; arrowhead in Fig. 2d). This asymmetric performance caused the position of both eyes to gradually but constantly shift with each successive motion cycle towards the side of lesion (orange dotted line, connecting peak responses in Fig. 2d). The directionally different dynamics of eye movement components during repetitive motion cycles was confirmed by evaluating the responses during the first half cycle in either direction starting from the resting table position (Fig. 3i, j). A typical example of directionally different eye movements immediately after the lesion (0 h) is illustrated in Fig. 3i, j. Peak-to-peak amplitudes and velocities of the eye movements in response to ipsilesionally directed head/table motion in darkness (Fig. 3i) dropped immediately after the nerve section (Fig. 3i, right) to rather negligible values, while eye movements evoked by head/table motion towards the intact (contralesional) side remained unaltered or even increased slightly in amplitude and dynamics compared to those recorded in controls (Fig. 3i, left).

Despite concurrent visuo-vestibular stimulation during the VOR in light, i.e. in the presence of a world-stationary vertical striped pattern, a considerable reduction of the response amplitude was observed, with a drop in gain from 0.32 ± 0.08 (mean ± SD; *n* = 6) in controls to a value of 0.22 ± 0.10 (mean ± SD; *n* = 6; Figs. 2e, 3d, g) immediately after the VIIIth nerve section (0 h). Even though vestibular-evoked eye movements in light appeared to be less asymmetric than in darkness, the reduction in response amplitude was similar for the VOR in darkness and in light. Thus, concurrently evoked visuo-motor responses during the VOR in light were unable to attenuate a full gain reduction immediately after the VIIIth nerve section; however, visual motion signals appeared to have allowed to at least partially offset the impaired eye motion dynamics during rotation in the ipsilesional direction. Nonetheless, eye movements evoked by head/table motion towards the ipsi- and contralesional (intact) side in light were as asymmetric in amplitude and dynamics (Fig. 3j) as those evoked during rotation in darkness (compare with left and right in Fig. 3i).

During the postlesional period, the VOR in darkness and in light continued to deteriorate further after the instantaneous recordings following the nerve section (0 h) to reach even lower values at 1.5 h postlesion. The gain of both VOR in darkness and in light dropped to 0.14 ± 0.10 (mean ± SD; *n* = 6; Fig. 3f, g), respectively. This further impairment derived largely from the eye motion component that was elicited during the stimulus half-cycle directed towards the ipsilesional side and was independent if a world-stationary visual pattern was present or not (compare plots in Fig. 3c, d). Most noticeably, however, the eye motion component during rotation towards the intact side also became considerably smaller at 1.5 h postlesion (cyan traces in Fig. 3c, d), suggesting a secondary effect as the origin for this severe impairment of the VOR gain. Despite the overall gain impairment, the response phase *re* stimulus position after the lesion remained largely unaltered at both time points for the VOR in darkness. In contrast, the smaller phase leads *re* stimulus position for the VOR in light after the transection of the VIII^th^ nerve remained and likely derived from a reweighted contribution of visual response components during rotation towards the ipsilesional side that were generally more in phase with the optokinetic stimulus (compare Fig. 3d, e; magenta and cyan traces). In addition, despite the loss of unilateral vestibular signals, the movements of both eyes were still highly conjugated (right plot in Fig. 2h; *λ* = 1.019; *r*^*2*^ = 0.8419; linear regression; *n* = 6), lending support to the decisive role of abducens internuclear neurons as substrate for this ocular motor behavior [19].

#### Impact on OKR performance

Immediately after the section of the VIIIth nerve (0 h), horizontal sinusoidal rotation of a black and white vertical striped visual pattern provoked typical phase-coupled oscillatory eye movements with similar dynamics, bilateral symmetry, phase relation and amplitude as those recorded before the lesion. This is illustrated by the eye movements over four cycles (Fig. 2f) as well as by the averaged responses over a single motion cycle (Fig. 3e). Statistical comparison confirmed the impression that the response gains immediately after the lesion (0 h) remained unaltered or even increased, although only slightly with respect to control values, to a mean gain of 0.14 ± 0.03 with a phase lag *re* stimulus of ~ 11° (10.6° ± 13.3°; mean ± SD; *n* = 6; Fig. 3h). In contrast and most surprisingly, the OKR gain severely deteriorated at 1.5 h postlesion to a magnitude of 0.07 ± 0.04 (mean ± SD; *n* = 6; Fig. 3h; Supplementary Fig. 2e), corresponding to a loss of 50% of the initial prelesional value. The severe reduction of the OKR gain at 1.5 h postlesion was unsuspected and comparable in magnitude to the overall gain reduction of the VOR in darkness and in light at the same postlesional time point. The delayed reduction in the amplitude of visuo-motor responses that were not immediately impaired by the unilateral lesion of the VIIIth nerve, but rather occurred only at a time point 1.5 h past the initial lesion, further corroborates the likely presence of extended secondary lesion-related effects. Plastic alterations in central areas which are potentially concerned with shared VOR-OKR circuit components, such as extraocular motoneurons or cerebellar elements, could be the *loci* of such a secondary effect.

### Amelioration or maintenance of VIIIth nerve lesion-induced deficits

Given the striking decrease in response performance 1.5 h postlesion, and to better evaluate the temporal progression of the ocular motor deficits, the performance of visuo-vestibular reflexes was further characterized at 3 and 4.5 h postlesion. These time points are often still inaccessible for a systematic evaluation of the VOR and OKR because of the slowly fading anesthesia and/or the presence of a roaring nystagmus in many animal species. At both time points (3 and 4.5 h postlesion) the number of “successful” attempts of the VOR in light and in darkness remained considerably lower than in controls (Supplementary Fig. 2a, b; Friedman test *p* = 0.2942 VOR in darkness, *p* = 0.6492 VOR in light), despite a slight, yet non-significant augmentation of “successful” episodes of the VOR in darkness at 4.5 h postlesion. In a complementary fashion, “unsuccessful” cycles of both visuo-vestibulo-motor responses after the VIIIth nerve section remained at an elevated frequency compared to controls (Supplementary Fig. 2a, b; Friedman test *p* = 0.0009 VOR in darkness, *p* = 0.4244 VOR in light), while the number of “fast phases” or eye movements designated “other” remained unaltered at all time points before and after the lesion (Supplementary Fig. 2a, b; Friedman test in darkness *p* = 0.6922 and in light *p* = 0.2537 for “fast phases”; *p* = 0.8781 in darkness and *p* = 0.4247 in light for “other”). This suggests again that the latter two categories of ocular motor behaviors are independent of vestibular activity.

In a corresponding manner, the respective gains of the VOR in darkness and in light as well as the OKR continued to remain severely depressed at 3 and 4.5 h postlesion (Supplementary Fig. 2c–e; Friedman test *p* = 0.0054 for VOR in darkness, *p* = 0.0041 for VOR in light; *p* = 0.0138 for OKR) with a tendency for a slight, yet non-significant amelioration at the latter time point. The phase of the responses remained largely unaltered (not shown) with the exception of those responses that included a visuo-motor component, that by definition was more in phase with stimulus position as already indicated by the control responses of the OKR (see above). The apparent slight amelioration of visuo-vestibular eye movements beginning at 4.5 h postlesion, however, resulted from a differential recovery of the ocular motor components during head/table motion in ipsi- and contralesional direction as indicated by the averages over a single motion cycle (see color-coded traces in Supplementary Fig. 1p–r). Accordingly, eye movements evoked by head/table motion towards the intact side, both in light and in darkness generated amplitudes and dynamics at 4.5 h postlesion that approached, though slowly, again those of prelesional controls (Supplementary Fig. 1p, q). In contrast, eye movements evoked by head/table rotation towards the ipsilesional side in light and in darkness continued to remain absent or had only very small amplitudes with very low dynamics (Supplementary Fig. 1p, q). These residual eye movements likely derived from a disfacilitation of the vestibular activity on the intact side during a head/table motion towards the ipsilesional side. The general amelioration after 4.5 h postlesion for vestibulo- and visuo-motor responses suggests that the improvement of eye motion magnitudes at this time point results from a gradually reestablished efficacy of cellular and circuit elements of the shared visuo-vestibulo-motor pathway.

## Discussion

Unilateral transection of the VIIIth nerve immediately provoked a severe impairment of the VOR in darkness with a smaller effect in light, which further deteriorated 1.5 h later to remain more or less unchanged for the next 3 h. In contrast, the OKR remained functionally intact and unaltered immediately after the vestibular loss (0 h) but experienced a considerable reduction starting at 1.5 h postlesion. The time course and occurrence of detrimental events at later time points suggest that the immediate impact by the loss of vestibular sensory signals is followed by secondary neuronal consequences involving shared VOR-OKR cellular and/or circuit components.

### Targeted transection of the VIIIth nerve and immediate impact on static eye position

The advantage of employing isolated head preparations of *Xenopus* tadpoles is the possibility to transect the VIIIth nerve under direct visual control, which thus ensures that other cranial nerves, the brainstem, or cerebellar structures remain entirely unaffected. Furthermore, bleeding or undesired tissue damage is completely avoided as it is potentially observed during comparable surgical interventions when performing a postganglionic neurectomy e.g. in mice [11]. Following control recordings, the section of the VIIIth nerve was complete and the preparation ready again for postlesional eye motion recordings within a few minutes, outcompeting other experimental models in terms of acute recordings. Moreover, the absence of any anesthetics during the surgical process in the isolated, yet functional, preparation circumvented all critical issues associated with anesthesia in an intact animal [11]. Most importantly, however, the employment of such an isolated preparation allowed an immediate evaluation of the impact of the unilateral vestibular loss on the behavioral consequences once the VIIIth nerve has been sectioned. This is not possible in any in vivo experiment to our knowledge, given the necessity to use anesthetics for the surgery and the difficulty to determine a clear time point when the effects of the anesthesia on neuronal activity have completely faded.

Immediately after the VIIIth nerve section in the current experiments, on average both eyes shift their resting position towards the side of the lesion, consistent with the imbalance in resting activity between the bilateral vestibular nuclei and the consequently asymmetric activation of extraocular motoneuronal pools [24, 25]. The lack of an acute spontaneous nystagmus after the unilateral vestibular lesion (see Fig. 2b) is likely an amphibian-specific particularity, related to the rather low resting activity in vestibular circuits in these animals [13, 26] and the corresponding small asymmetry of bilateral vestibular resting rates. The relatively variable shift in resting eye position between different preparations might be related to the specific magnitude of the bilateral vestibular firing rate asymmetry after the lesion and/or reflect a dependency of the postlesional shift on the prelesional eye position as suggested from the data plotted in Fig. 2c. In correspondence, the presence of a spontaneous nystagmus in mammalian species [27–29] is likely related to larger bilateral vestibular firing rate asymmetries after a unilateral loss due to generally higher vestibular discharge rates in these animals.

### Acute impact of VIIIth nerve transection on visuo-vestibular motion-evoked eye movements

Immediately after the VIIIth nerve section (0 h), the gain of the horizontal angular VOR in darkness decreased by ~ 30% (Fig. 3f, g). This instantaneous deterioration directly derives from the disconnection of the respective semicircular canal signals on the ipsilesional side, which as expected, substantially contribute to the excitatory drive for extraocular motoneurons. A gain reduction of the VOR in light is also noticeable even though vestibular and visual motion signals are concurrently activated during horizonal head rotation in the presence of a world-stationary vertical black and white striped pattern. This suggests that simultaneously activated visuo-motor reflexes are unable to acutely substitute the unilaterally disconnected vestibular signals. This also complies with the finding that the gain of the OKR remained largely unaltered immediately after the VIIIth nerve section (0 h; Figs. 2f, 3h). Following the initial reduction of VOR amplitudes, response gains showed no recovery but instead deteriorated further over the first few hours after the nerve section to remain low, in compliance with the many reports indicating severely impaired eye movements for many days after the lesion [11, 22, 25, 28].

Unexpectedly, however, the performance of the OKR at 1.5 h postlesion was also severely impaired (Fig. 3e, h) with a gain reduction by ~ 50% comparable to the impairment of the VOR at this time point. This delayed deterioration of the OKR is surprising because the underlying short-latency direct pathway, known to mediate this reflex in amphibians [30], was per se not affected by the vestibular lesion. The severe reduction in OKR performance along with the equally drastic impairment of the VOR at 1.5 h postlesion could, however, result from an initiation of a secondary effect by the ongoing bilateral asymmetric neuronal activity in vestibulo-motor circuits. The resulting persistence of higher firing rates in vestibular circuits on the intact side after the lesion, reinforced by commissural inhibitory connections [31], may have induced a homeostatic plasticity [32] that caused a reduction of the respective synaptic gains within the shared OKR-VOR circuit elements. Such an adaptive process has, in fact, been described for extraocular motor discharge of *Xenopus* tadpoles after continuous (> 20 min) excessive sinusoidal vestibular motion stimulation [33]. This plasticity depended on an intact cerebellum and caused an attenuation of the extraocular motor output. Because cerebellar circuits integrate vestibular inputs and residual retinal image slip signals to adequately adjust eye motion magnitudes [34], the vestibular imbalance after a unilateral VIIIth nerve lesion might have also prompted the circuitry to down-regulate the gains of the ocular motor output during both OKR and VOR.

While homeostatic plasticity processes are known to occur during long-term recalibration of vestibular deficits [25, 35], such plasticity processes could also be triggered during the initial phase after a unilateral vestibular lesion. This assumption allows the generation of testable hypotheses, which probe the possibility that the impairment of vestibulo-motor reflexes is a combinatorial effect of the unilateral loss of vestibular signals followed by an attenuation of central vestibulo-motor signal processing as part of an adaptive plasticity response, prior to and even unrelated to the process of “vestibular compensation”. Comparison of the outcome of studies when the animals remain in darkness between the recordings (current study), or in light with a stationary striped pattern providing a continuous visual reference allows testing the hypothesis that the delayed deterioration of visuo-motor reflexes is a result of an ongoing homeostatic plasticity after a VIIIth nerve section. Moreover, gaze stabilization by visuo-vestibular sensory signals is supplemented in most vertebrates by neck/body/limb proprioceptive signals [34] and even supplanted by spinal locomotor efference copies in amphibians [20]. While it is known that the contribution of such signals to gaze stabilization collectively increases at the extended, chronic, time period after a VIIIth nerve lesion [36], it would be highly interesting to test the efficacy of the respective ocular motor responses immediately after the lesion. Accordingly, recordings of eye movements during fictive locomotion in *Xenopus* tadpoles [20] before and after transection of the VIIIth nerve would reveal if the respective ocular motor performance is also subjected to a delayed gain diminishment as shown for the OKR. Alternatively, locomotor efference copy-evoked eye movements might remain unaltered after the lesion, potentially because of the direct pathway connections between the spinal central pattern generator and extraocular motoneurons, which bypass central vestibular nuclei and the cerebellum [20].

### Clinical implications

The current findings in *Xenopus* tadpoles specifically highlight the fact that the spectrum of observed symptoms after a unilateral peripheral vestibular lesion [28, 29, 37] might not exclusively reflect the bilateral imbalance in resting activity of the vestibular circuitry. Rather, the observed static and dynamic syndromes, also present in human patients, could be a combinatorial effect that results from the sudden unilateral loss of peripheral vestibular bulk discharge and a secondary consequence that causes an extreme form of housekeeping-related plasticity reactions, which normally aim at consolidating the synaptic gain at a preset value [32]. Such a process would assist the initial step of restituting the excessive activity. The asymmetric activity is integrated and interpreted as a single ongoing motion percept. As a resultant consequence, the synaptic efficacy along shared visuo-vestibulo-motor pathways is reduced and eye movement magnitudes are attenuated, despite being behaviorally inappropriate. Even though this hypothesis derived from results in amphibians without a roaring nystagmus after the VIIIth nerve lesion, it is likely that a comparable neuronal plasticity is also induced in mammals, including humans. In fact, the generally higher vestibular resting activity in mammalian species [34] provokes an even larger bilateral asymmetry after a unilateral loss of peripheral sensory inputs. Such a pathophysiological condition is interpreted as constant rotation towards the intact side, hence the nystagmus in mammals, which as a consequence should also trigger a diminishment of the gain because of the continuous excessive motion signaling in the vestibular system (see [33]). Unilateral VIIIth nerve section in mammals thus creates an even larger necessity to diminish the excessive activity by a homeostatic mechanism. Therefore, differences in the immediate consequences of a VIIIth nerve lesion between amphibians and mammals should be quantitative rather than qualitative. Provided that this assumption is correct, an immediate reduction of the initial asymmetric vestibular activity after a planned surgery in humans through, for example, hyperpolarizing galvanic vestibular stimulation of the intact side [38] or by directionally appropriate constant velocity visual motion stimulation might be beneficial for a faster recovery from the static deficits after a peripheral vestibular lesion. Visual image motion, and in particular in the framework of the OKR, play, in fact, an important role for gaze stabilization and motion perception given the integration with vestibular signals under normal conditions, and even more so under pathophysiological conditions (e.g. [39]). After the acute phase of a unilateral vestibular loss, visual motion has been employed in rehabilitation treatments of vestibular patients [39, 40], and such motion signals are likely also part of a general sensory substitution strategy, potentially leading to a long-term increase in the contribution and magnitude of the OKR to image stabilization [4, 5, 7].

## Electronic supplementary material

Below is the link to the electronic supplementary material.

** Supplementary Fig. 1 **Visuo-vestibular motion-evoked eye movements before and after a section of the VIIIth nerve. **a-o** Average responses (color-coded solid lines) ± SD (color-coded areas) over a single cycle of sinusoidal head/table motion in darkness (VOR in darkness), in light (VOR in light) and visual pattern motion (OKR) separately before the lesion (**a-c**), immediately after (0 hours; **d-f**), at 1.5 hours (**g-i**), 3 hours (**j-l**) and 4.5 hours postlesion (**m-o**) from all recorded preparations (*n* = 6), respectively; dashed sinusoids represent stimulus position profiles (S_pos_); note that the stimulus frequency and peak velocity for head/table motion is 0.5 Hz and 31.4°/s and for the visual pattern motion 0.2 Hz and 12.6°/s. **p-r** Overlay of average responses over single motion cycles shown in **a-o** from all time points (SDs were omitted) (EPS 9691 kb)

**Supplementary Fig. 2 **Proportional distribution and performance of motion-evoked eye movements before and after a section of the VIIIth nerve. **a, b** Relative proportions of the categories “successful”, “unsuccessful”, “fast phases” and “other” during head/table motion in darkness (VOR in darkness, **a**) and in light (VOR in light, **b**) before the lesion, immediately after (0 hours) and at 1.5 hours, 3 hours and 4.5 hours postlesion. **c-e** Progression of the response gain (eye motion / stimulus motion) before the lesion, immediately after (0 hours) and at 1.5 hours, 3 hours and 4.5 hours postlesion for the VOR in darkness (**c**), in light (**d**) and OKR (**e**); the time points in all plots are color-coded and specified in the right upper corner of **a** (EPS 755 kb)
